# Improving the Diagnosis of Bacterial Infections: Evaluation of 16S rRNA Nanopore Metagenomics in Culture-Negative Samples

**DOI:** 10.3389/fmicb.2022.943441

**Published:** 2022-07-14

**Authors:** Coralie Bouchiat, Christophe Ginevra, Yvonne Benito, Tiphaine Gaillard, Hélène Salord, Olivier Dauwalder, Frédéric Laurent, François Vandenesch

**Affiliations:** ^1^Laboratoire de Bactériologie, Institut des Agents Infectieux, Biologie Moléculaire, Hospices Civils de Lyon, Lyon, France; ^2^Equipe Inserm U1111, Centre International de Recherche en Infectiologie, Université de Lyon, Université Claude Bernard Lyon 1, CNRS, UMR5308, ENS de Lyon, Lyon, France; ^3^Centre National de Référence des Légionelles, Institut des Agents Infectieux, Hospices Civils de Lyon, Lyon, France; ^4^Laboratoire de biologie médicale, Hôpital d'Instruction des Armées Desgenettes, Service de Santé des Armées, Lyon, France

**Keywords:** metagenomics, bacterial infections, molecular diagnosis, Nanopore sequencing, 16S rRNA

## Abstract

While 16S rRNA PCR-Sanger sequencing has paved the way for the diagnosis of culture-negative bacterial infections, it does not provide the composition of polymicrobial infections. We aimed to evaluate the performance of the Nanopore-based 16S rRNA metagenomic approach, using both partial and full-length amplification of the gene, and to explore its feasibility and suitability as a routine diagnostic tool for bacterial infections in a clinical laboratory. Thirty-one culture-negative clinical samples from mono- and polymicrobial infections based on Sanger-sequencing results were sequenced on MinION using both the in-house partial amplification and the Nanopore dedicated kit for the full-length amplification of the 16S rRNA gene. Contamination, background noise definition, bacterial identification, and time-effectiveness issues were addressed. Cost optimization was also investigated with the miniaturized version of the flow cell (Flongle). The partial 16S approach had a greater sensitivity compared to the full-length kit that detected bacterial DNA in only 24/31 (77.4%) samples. Setting a threshold of 1% of total reads overcame the background noise issue and eased the interpretation of clinical samples. Results were obtained within 1 day, discriminated polymicrobial samples, and gave accurate bacterial identifications compared to Sanger-based results. We also found that multiplexing and using Flongle flow cells was a cost-effective option. The results confirm that Nanopore technology is user-friendly as well as cost- and time-effective. They also indicate that 16S rRNA targeted metagenomics is a suitable approach to be implemented for the routine diagnosis of culture-negative samples in clinical laboratories.

## Introduction

Rapid and accurate identification of the etiological agent of infection is the cornerstone of appropriate antimicrobial therapy and its successful management. While bacterial culture is the gold standard for microbiological diagnosis, it can be undermined and remain sterile in case of antimicrobial treatment prior to sampling, an infection caused by fastidious agents, or other viable but non-culturable (VBNC) microorganisms (Fenollar and Raoult, 2004; Laupland and Valiquette, 2013). Moreover, the typical turnaround time of 24–48 h for cultures can be extended to 7–14 days when slow-growing agents are involved. Molecular microbiology has helped overcome such limitations by providing bacterial identification in <24 h, enabling early-targeted therapy (Fenollar and Raoult, 2004; Renvoisé et al., 2013; Trotter et al., 2019). Specifically, 16S rRNA PCR has high sensitivity and specificity and is proven to be a powerful strategy for bacterial infection diagnosis in case of the absence of etiological orientation by broad-range DNA amplification (Fenollar et al., 2006; Renvoisé et al., 2013). The amplification of the 16S rRNA gene can cover all or some of the nine hypervariable regions scattered among highly conserved sequences. Ensuing sequencing of the amplicons and its comparison to nucleotide database allows assignment to a taxum. However, molecular diagnosis by conventional Sanger sequencing of 16S rRNA amplicons is hampered in case of a polybacterial infection. Metagenomic next-generation sequencing from biological samples has paved the way for a paradigm shift in microbiological diagnosis without *a priori* etiological assumption (Goodwin et al., 2016; Parize et al., 2017). This approach overcomes the shortcomings of both culture and PCR, by combining speed with comprehensive coverage of all the different microorganisms present in the sample. Nonetheless, the cost, complexity, and time needed for sample preparation, along with the bioinformatics, remained a pitfall in the deployment of such technology on a routine basis (Goodwin et al., 2016). Nanopore sequencing technology allows rapid and user-friendly library preparation, real-time data acquisition, and de-skilled and turnkey data analysis through an online platform with dedicated pipelines (Goodwin et al., 2016; Kai et al., 2019; Moon et al., 2019). It can be used either with regular flow cells or with the miniaturized cheaper version, the Flongle. Multiple studies have evaluated the gain in the information of both the 16S rRNA targeted and non-targeted metagenomic approaches, specifically in high pathogen load samples, such as respiratory infections, and its concordance with culture-positive samples (Mitsuhashi et al., 2017; Moon et al., 2017; Ashikawa et al., 2018; Charalampous et al., 2019; Dyrhovden et al., 2019; Hong et al., 2019; Kai et al., 2019; Yang et al., 2019; Chan et al., 2020; Leggett et al., 2020). However, little is known about the performance of the 16S rRNA-targeted approach on negative-culture samples and therefore no diagnosis (Moon et al., 2017, 2019; Cheng et al., 2018; Kai et al., 2019; Wang et al., 2020). Moreover, several issues remain to be addressed such as the risk of contamination, dealing with background noise, bacterial identification accuracy, and ensuing clinical-biological interpretation (Street et al., 2017; Sanderson et al., 2018).

Therefore, we aimed to evaluate the use of 16S rRNA Nanopore metagenomics as a routine diagnostic tool in negative-culture bacterial infections. We first addressed the risk of contamination, the definition of background noise, and bacterial identification accuracy. Performance was then compared between the in-house partial 16S rRNA PCR amplification and the Nanopore full-length 16S rRNA metagenomic kit; time-effectiveness and cost optimization were also evaluated.

## Materials and Methods

### Clinical Samples

Upon clinical suspicion of bacterial infection, amplification and Sanger-based sequencing of a portion of the 16S rRNA gene is routinely performed in our laboratory on culture-negative difficult-to-obtain samples such as bone and joint specimens, cardiac valve, brain abscesses, etc., as previously described (Gauduchon et al., 2003). Around 400 culture-negative and 16S rRNA, PCR-positive samples are obtained annually, of which 25% lead to unsuccessful Sanger sequencing. In the present study, we retrospectively searched the laboratory database for culture-negative and 16S rRNA PCR-positive samples with either a single DNA sequence or an uninterpretable polybacterial pattern chromatogram obtained from patients admitted between January and July 2019. The sample localisation and basic clinical context that came with the bacteriology analysis prescription were collected when available.

### DNA Preparation and Sequencing

**Figure 1:**
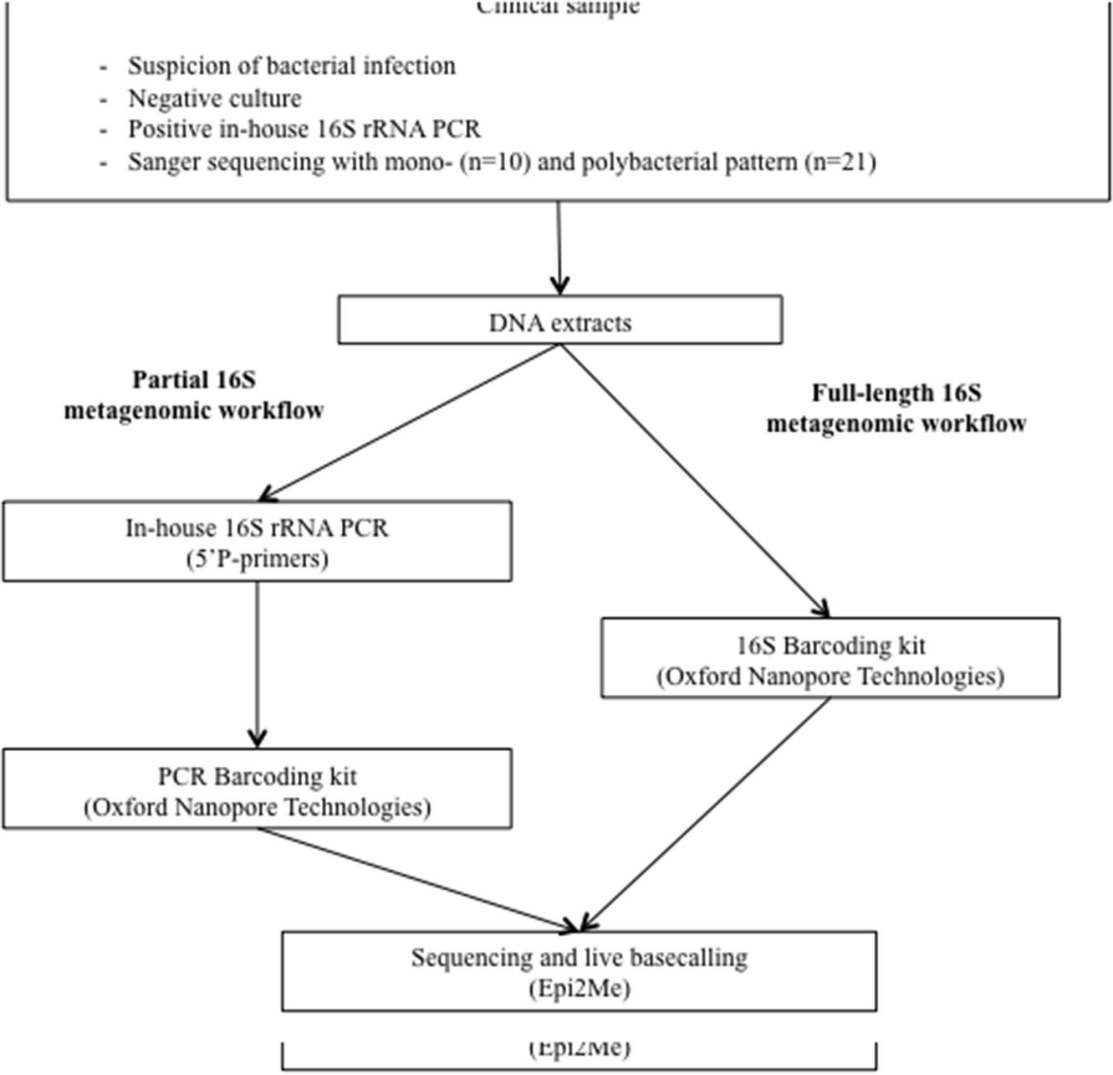
Study design. On a routine basis, culture-negative clinical samples for which infection is suspected are sent to the molecular diagnosis platform for an in-house amplification of a portion of the 16S rRNA gene followed by Sanger-based sequencing. In the present study, DNA extracts were re-tested for (i) partial and, (ii) full-length 16S rRNA metagenomic analysis with Nanopore technology.

Remnant DNA extracts of the included samples were retrieved from −20°C storage in April–June 2020. Nanopore metagenomic analysis was performed using PCR products from both the V6/V8 region and full-length amplification of the 16S rRNA gene. Briefly, the in-house approach consists of the amplification of the V6/V8 portion of the 16S rRNA gene followed by barcoding of the amplicons with a dedicated Nanopore kit, while the full-length protocol consists of a one-step amplification and barcoding with another dedicated Nanopore kit (Figure 1).

#### In-House Partial (V6/V8) 16S rRNA Gene Nanopore Metagenomic Protocol

In-house partial (480 bp) 16S rRNA PCR was performed using the 91E and 13BS 5′-phosphorylated primers, as previously described (Gauduchon et al., 2003), and run for 40 cycles. Libraries were then prepared using the PCR Barcoding kit (SQK-PBK004; Oxford Nanopore Technologies, Oxford, UK) according to the manufacturer's protocol (version PBK_9073_v1_revF_23May2018) optimized as follows: phosphorylated 16S amplicons were directly purified using solid-phase reversible immobilization (SPRI) beads (NucleoMag NGS Clean-up and size selected, Macherey-Nagel, Hoerdt, France; 1.2×, 5 min), followed by PCR adapter ligation (Blunt/TA Ligase Master Mix; New England Biolabs, Evry, France), and an additional SPRI bead purification (1×, 5 min). After the barcoding PCR (LongAmp Taq 2× Master Mix, New England Biolabs), a final SPRI bead purification was performed (2×, 10 min).

#### Full-Length 16S rRNA Gene Nanopore Metagenomic Protocol

Samples were prepared using the 16S Barcoding kit (SQK-RAB204; Oxford Nanopore Technologies) according to the manufacturer's protocol (version RAB_9053_v1_revL_14Aug19) except that the PCR program was changed to a run of 45 cycles instead of 25 to increase its sensitivity.

#### Nanopore Sequencing

After the quantification of purified DNA barcoded amplicons (Qubit dsDNA HS Assay kit, Thermo Fischer Scientific, Illkirch-Graffenstaden, France), 60–80 fmol of sequencing library DNA were loaded into a regular flow cell (FLO-MIN 106D R9.4.1, Oxford Nanopore Technologies) or 20 fmol into its smaller version, the Flongle flow cell (FLO-FLG 001; Oxford Nanopore Technologies). Each sequencing run included a negative control consisting of PCR-grade water that was introduced at the 16S rRNA amplification step and underwent the full process.

### Sequencing Run and Data Analysis

Sequencing runs on the MinION device were driven by the MinKNOW software (version 3.6.5, Oxford Nanopore Technologies) to allow raw data acquisition and live base calling. Sequencing FASTQ reads were analyzed in real-time using the cloud-based platform EPI2ME Agent (v2020.2.10; Metrichor, Oxford, UK) using the 16S workflow. This turnkey tool includes quality-check, demultiplexing, primary and secondary analyses to obtain an identification of bacteria and archaea and an NCBI-based taxonomy tree for each sample.

### Time-Effectiveness Evaluation and Cost Optimization

The total amount of reagents required for one run was calculated in euros for each protocol and for both the regular flow cell and the Flongle and divided by the number of clinical samples tested in the associated run. The duration of each approach was estimated step-by-step based on observed times. Labour cost was not included since it can be very variable between countries. Cost-optimized runs on Flongle were performed with clinical samples 1–5.

### Ethics Committee Approval

This project was conducted according to the French law in force at the time of the study and ethical principles outlined in the Declaration of Helsinki. It obtained approval by the institutional ethics committee Hospices Civils de Lyon—CNIL (number 20-265).

## Results

The 31 clinical samples originated from bone and joint specimens (bone, *n* = 10; tissue, *n* = 4; joint fluid, *n* = 2; reaming product, *n* = 1; abscess, *n* =1), pleural aspirates (*n* = 3), aqueous humour (*n* = 2), cardiac valves (*n* = 2), abscesses (brain, *n* = 1; liver, *n* = 1; spleen, *n* = 1), and tissue biopsies (vascular, n = 1; soft tissue, *n* = 2).

### Overcoming the Contamination and Background Noise Pitfalls by Setting a Threshold

Using the partial 16S approach with regular flow cells, the 31 clinical samples were sequenced in three consecutive runs, in which negative controls yielded 1–15 bacterial reads, accounting for < 0.001–0.2% of the total number of reads in a run. None of these bacterial reads met the predominant bacteria detected in the samples of the associated run, ruling out the hypothesis of cross-contamination of samples or reagent contamination. Bacterial reads from the clinical samples that were present in a lower number than in the associated negative control were not taken into account for further analysis.

**Figure 2:**
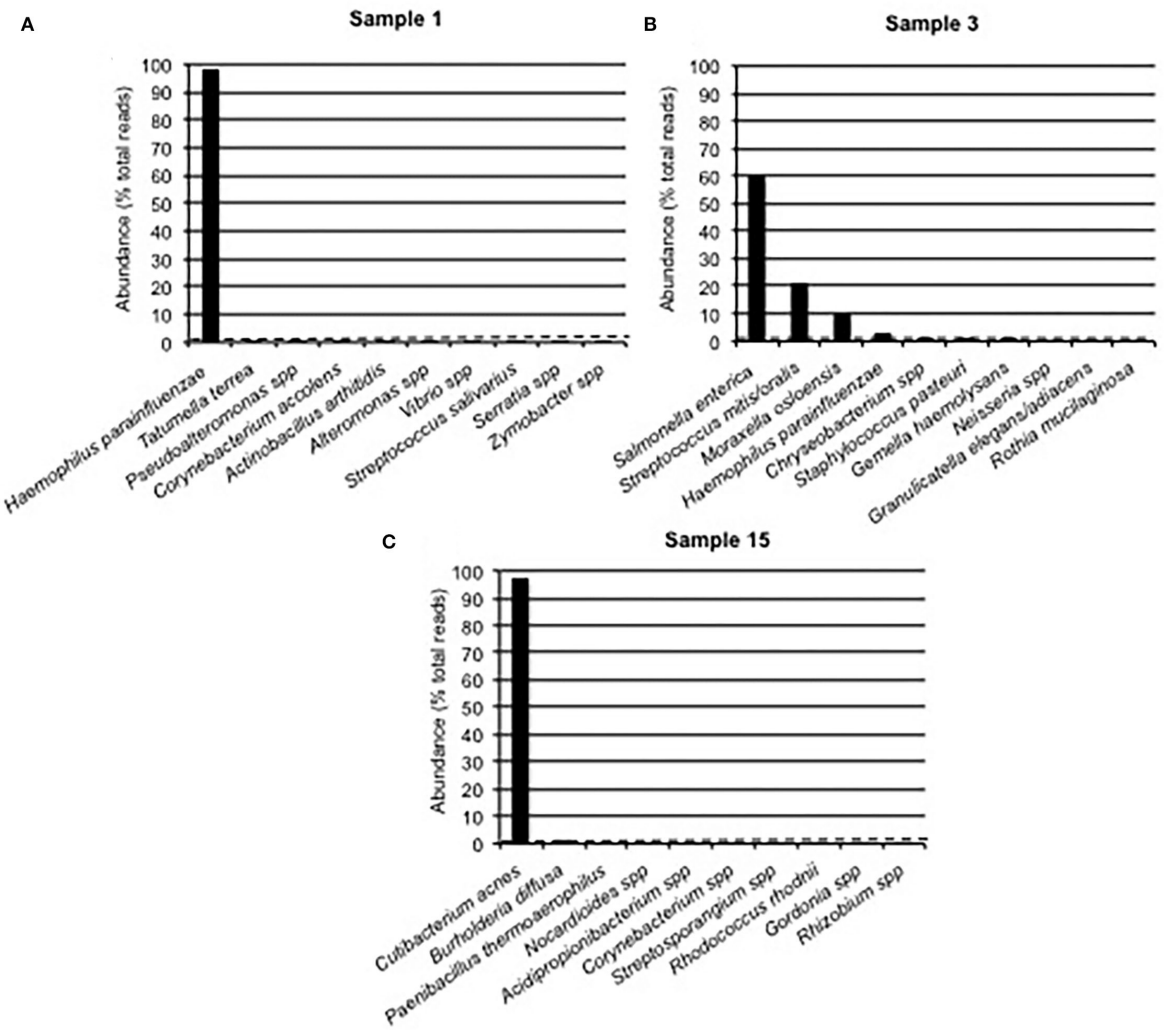
The Bacterial Composition of Clinical Samples Is Based on Nanopore Technology. Examples of Partial 16S Approach Results From Sanger-Based Mono- and Polymicrobial Clinical Samples. An Abundance Histogram Represents the Proportion of Reads Assigned to up to 10 Predominant Bacteria. The Dotted Line Displays the 1% Abundance cut-off. **(A)** The Presence of one Single Bacterium Above the 1% cut-off, Followed by a Steep Fall Distribution of Other Detected Bacteria, Concluded to a Monomicrobial Sample With Some Background Noise. **(B)** The Presence of More Than one Bacterium Above the 1% cut-off Concluded to a Polymicrobial Sample, Along With Some Background Noise. **(C)** Sample 15 Displayed the Presence of 2 Bacteria Above the 1% cut-off, While Their Respective Abundances Were Greatly Different.

We used the results of the Sanger-based monobacterial infections (samples 1–10) as an estimate of background signals. Among them, nine [samples 1 (Figure 2A), 2, and 4–10] revealed the presence of one predominant bacterium; corresponding reads accounted for 97.05–99.65% of all reads (Supplementary Table 1). For each of these nine samples, the rest of the reads were assigned to a mean of 45 different bacteria (range: 6–96), with an infinitesimal (< 0.01–0.97%) presence of each, corresponding to an irrelevant signal phenomenon. Setting a threshold value of at least 1% of all reads defined the background noise. The last sample (number 3) had a first-ranked bacterium accounting for 60.28% of all reads, followed by five other bacteria above the 1% abundance cut-off, which revealed its polybacterial nature (Figure 2B).

Of the 21 samples with a Sanger polybacterial pattern chromatogram, 4 (samples 11, 13, 14, and 16) revealed the presence of the first-ranked bacterium with an abundance of 96.50–99.26% followed by multiple bacteria with an abundance below the 1% threshold (0.2–0.9%; Supplementary Table 1) and were thus considered monomicrobial. Two other samples (samples 12 and 15) had a predominant bacterium with an abundance of 97.56 and 97.08%, respectively, followed by a second-ranked bacterium whose abundance was considerably lower (1.19 and 1.79%, respectively), while just above the 1% cut-off value (Supplementary Table 1, Figure 2C). The remaining 15 samples revealed the presence of at least three bacteria above the determined cut-off and were then categorized as polymicrobial samples (Supplementary Table 1). Noticeably, in sample 31 (pleural aspirate), *Parvimonas micra* and *Mogibacterium diversum* were considered as background noise due to a <1% abundance. Conversely, in sample 22 (aortic valve) six bacteria (*Cutibacterium acnes, Enterobacter cloacae, Moraxella* spp., *S. mitis/oralis, Stenotrophomonas pavanii*, and *Chryseobacterium* spp.) were >1% and considered notable.

### Contribution of Nanopore 16S rRNA Targeted Metagenomics to Routine Diagnosis

All samples determined to be monomicrobial by Sanger sequencing were also determined to be so by Nanopore sequencing using the in-house partial 16S approach (Table 1). Regarding the 21 Sanger-based polymicrobial samples, Nanopore technology, and the partial 16S approach found that four of them were monomicrobial (samples 11, 13, 14, and 16) including *Streptococcus dysgalactiae* in a bone and joint infection (sample 11) and *Raoultella* spp. in an endophtalmitis (sample 16). Among the 17 remaining samples that were categorized as polymicrobial, Nanopore sequencing allowed the identification of the composing bacteria; for example, *Porphyromonas gingivalis, Fusobacterium nucleatum, Tannerella forsythia, Filifactor alocis*, and *Staphylococcus aureus/simiae/haemolyticus* in an infected mandible fragment (sample 29). Likewise, sample 31, a pleural aspirate, was found to be composed of *Fusobaterium nucleatum, Treponema lecithinolyticum, Porphyromonas gingivalis*, and *Tannerella forsythia* by the in-house partial 16S Nanopore approach, suggesting oropharyngeal flora; and sample 28, from a brain abscess, led to the identification of the *Schaalia meyeri, Fusobacterium nucleatum, Campylobacter rectus, Neisseria artica, Kingella denitrificans*, and *Actinomyces israelii* (Supplementary Table 1).

**Table 1:** Characteristics and bacterial identifications based on Sanger sequencing, full-length 16S rRNA Nanopore sequencing, and in-house partial 16S rRNA Nanopore sequencing.

**Sample ID**	**Sample type**	**Clinical context**	**Sanger-based sequencing ID**	**Nanopore-based sequencing ID (top 10 bacteria with** **>1% abundance)**		
				**Full-length 16S rRNA (sorted by decreasing abundance)**	**In-house partial 16S rRNA (sorted by decreasing abundance)**	
				**MinION regular flow-cell run**	**MinION regular flow-cell run**	**MinION Flongle run**
1	Bone	NA	*Haemophilus parainfluenzae*	*Haemophilus parainfluenzae*	*Haemophilus parainfluenzae*	*Haemophilus parainfluenzae*
2	Bone	Chronic bone abscess	*Clostridium* spp.	*Clostridium massilodielmoense*	*Clostridium massilodielmoense*	*Clostridium massilodielmoense*
3	Bone	Chronic osteomyelitis	*Enterobacteriaceae*	No amplification	*Salmonella enterica*	*Salmonella enterica*
					*Streptococcus mitis/oralis/* *pseudopneumoniae*	*Streptococcus oralis/* *pseudopneumoniae*
					*Moraxella osloensis*	*Moraxella osloensis*
					*Haemophilus parainfluenzae*	*Haemophilus parainfluenzae*
					*Chryseobacterium* spp.	*Chryseobacterium* spp.
					*Staphylococcus pasteuri*	*Staphylococcus pasteuri*
4	Bone abscess	Sarcoma	*Bacillus* spp.	No amplification	*Bacillus mobilis*	*Bacillus mobilis*
5	Bone	Hip prosthesis infection	*Klebsiella pneumoniae*	No amplification	*Klebsiella pneumoniae*	*Klebsiella pneumoniae*
6	Aortic valve	Infective endocarditis	*Streptococcus gallolyticus*	*Streptococcus gallolyticus*	*Streptococcus gallolyticus*	Not tested
7	Bone	Tibial external fixators infection	*Staphylococcus* spp.	*Staphylococcus aureus/lugdunensis/haemolyticus*	*Staphylococcus aureus/lugdunensis/simiae*	Not tested
8	Aqueous humour	Post cataract-surgery endophtalmitis	*Streptococcus* spp.	*Streptococcus mitis/oralis/pneumonia/* *pseudopneumoniae*	*Streptococcus oralis/himalayensis/* *parasanguinis/pneumoniae*	Not tested
9	Liver abscess	Liver abscess returning from Algeria trip	*Klebsiella* spp.	*Klebsiella pneumoniae*	*Klebsiella* spp.	Not tested
10	Pleural aspirate	Pleuropneumonia	*Streptococcus pyogenes*	*Streptococcus pyogenes*	*Streptococcus pyogenes*	Not tested
11	Tissue from bone and joint infection	NA	Polybacterial pattern chromatogram	No amplification	*Streptococcus dysgalactiae*	Not tested
12	Soft tissue biopsy	NA	Polybacterial pattern chromatogram	*Streptococcus agalactiae*	*Streptococcus agalactiae*	Not tested
					*Porphyromonas endodontalis*	
13	Disc biopsy	Spondylodiscitis	Polybacterial pattern chromatogram (possible presence of *Staphylocococcus* spp)	*Staphylococcus epidermidis/capitis/saccharolyticus*	*Staphylococcus epidermidis/saccharolyticus*	Not tested
14	Elbow joint fluid	NA	Polybacterial pattern chromatogram	*Staphylococcus aureus*	*Staphylococcus simiae/aureus/haemolyticus*	Not tested
15	Reaming product	Hip prosthesis infection	Polybacterial pattern chromatogram	No amplification	*Cutibacterium acnes*	Not tested
					*Burholderia diffusa*	
				**Full-length 16S rRNA (sorted by decreasing abundance)**	**In-house partial 16S rRNA (sorted by decreasing abundance)**	
				**MinION regular flow-cell run**	**MinION regular flow-cell run**	**MinION Flongle run**
16	Aqueous humour	Endophtalmitis	Polybacterial pattern chromatogram	No amplification	*Raoultella* spp.	Not tested
17	Joint fluid	NA	Polybacterial pattern chromatogram	*Acinetobacter parvus/septicus*	*Chryseobacterium* spp.	Not tested
				*Moraxella osloensis*	*Acinetobacter parvus/septicus*	
				*Paracoccus lutimaris*	*Paracoccus lutimaris*	
				*Chryseobacterium hominis*	*Moraxella osloensis*	
				*Pseudoxanthomonas* spp.	*Microbacterium lacticum*	
				*Xanthomonas* spp.	*Haematobacter massiliensis*	
					*Pseudoxanthomonas* spp. spp.	
18	Bone	Sacroileitis	Polybacterial pattern chromatogram	*Streptococcus parasanguinis/salivarius*	*Porphyromonas endodontalis/gingivalis*,	Not tested
				*Porphyromonas endodontalis/gingivalis*	*Streptococcus pseudopneumoniae/* *parasanguinis*	
				*Peptinophilus* spp.	*Staphylococcus hominis/epidermidis/* *saccharolyticus*,	
				*Veillonella ratii/tobetsuensis Cutibacterium acnes*	*Veillonella ratti/tobetsuensis*,	
				*Anaerococcus nagyae*	*Corynebacterium lipophiloflavum/pilbarense*,	
				*Staphylococcus saccharolyticus*	*Micrococcus* spp.	
				*Prevotella salivae*	*Cutibacterium* spp.	
				*Finegoldia magna*	*Dermacoccus* spp.	
				*Rothia mucilaginosa*	*Rothia mucilaginosa*	
					*Finegoldia magna*	
19	Soft tissue biopsy	Deep-seated soft tissue infection	Mixed chromatogram	No amplification	*Corynebacterium kroppenstedtii/* *tuberculostearicum*	Not tested
					*Morganelle morganii*	
					*Anaerococcus octavus/nagyae*	
					*Cutibacterium acnes*	
					*Staphylococcus epidermidis/pasteurii/capitis*	
					*Prevotella veroralis/maculosa*	
					*Streptococcus parasanguinis/gordonii*	
					*Actinomyces oris*	
					*Finegoldia magna*	
					*Stenotrophomonas koreensis*	
20	Thorax tissular biopsy	Firearm injury	Mixed chromatogram	*Oscillibacter valericigenes*	*Bacteroides uniformis/vulgatus*	Not tested
				**Full-length 16S rRNA (sorted by decreasing abundance)**	**In-house partial 16S rRNA (sorted by decreasing abundance)**	
				**MinION regular flow-cell run**	**MinION regular flow-cell run**	**MinION Flongle run**
				*Bacteroides uniformis/vulgatus*	*Bifidobacterium* spp.	
				*Ruminococcus* spp.	*Oscillibacter valericigenes*	
				*Dialister invisus*	*Collinsella aerofaciens*	
				*Paenibacillus* spp.	*Paenibacillus* spp.	
				*Hungateiclostridium* spp.	*Ruminococcus* spp.	
				*Romboutsia timonensis*	*Hungateiclostridium* spp.	
				*Ruminococcus* spp.	*Dialister invisus*	
				*Clostridium saudiense*	*Parvimonas micra*	
					*Alistipes putredinis*	
21	Pleural aspirate	Pleuropneumonia	Mixed chromatogram	*Filifactor alocis*	*Porphyromonas gingivalis*	Not tested
				*Fusobacterium nucleatum*	*Treponema maltophilum/denticola/medium*	
				*Mogibacterium timidum*	*Fusobacterium nucleatum*	
				*Porphyromonas gingivalis*	*Filifactor alocis*	
				*Treponema maltophilum/denticola/medium*	*Tannerella forsythia*	
				*Tannerella forsythia*	*Schaalia cardiffensis*	
				*Peptococcus niger*	*Mogibacterium timidum*	
				*Prevotella seregens*	*Prevotella seregens*	
22	Aortic valve	Infective endocarditis	Mixed chromatogram	*Cutibacterium acnes*	*Cutibacterium acnes*	Not tested
				*Streptococcus mitis/oralis*	*Enterobacter asburiae/cloacae*	
				*Enterobacter asburiae/cloacae*	*Stenotrophomonas pavanii/hibiscicola*	
				*Stenotrophomonas pavanii/hibiscicola*	*Chryseobacterium* spp.	
					*Moraxella* spp.	
					*Streptococcus mitis/oralis*	
23	Vascular tissue	Vascular prosthesis infection in a context of lumbosacral eschar	Mixed chromatogram	*Acinetobacter junii*	*Cloacibacterium normanense*	Not tested
				*Pseudomonas stutzerii*	*Moraxella osloensis*	
				*Aeromonas schubertii*	*Prevotella* spp.	
				*Anoxybacillus* spp.	*Pleomorphomonas oryzae*	
				*Aquabacterium* spp.	*Tolumonas osonensis*	
					*Aeromonas schubertii*	
					*Bacteroides graminisolvens*	
					*Geobacillus stearothermophilus*	
					*Acinetobacter johnsonii*	
					*Brachybacterium paraconglomeratum*	
				**Full-length 16S rRNA (sorted by decreasing abundance)**	**In-house partial 16S rRNA (sorted by decreasing abundance)**	
				**MinION regular flow-cell run**	**MinION regular flow-cell run**	**MinION Flongle run**
24	Spleen abscess	NA	Mixed chromatogram	*Porphyromonas endodontalis*	*Bacteroides fragilis*	Not tested
				*Bacteroides fragilis*	*Porphyromonas endodontalis*	
				*Campylobacter rectus/gracilis*	*Streptococcus* spp.	
				*Fusobacterium nucleatum*	*Treponema lecithinolyticum*	
				*Treponema lecithinolyticum*	*Haemophilus parainfluenzae*	
					*Bacillus mobilis*	
					*Klebsiella pneumoniae*	
					*Staphylococcus epidermidis/haeomolyticus*	
25	Tissue from bone and joint infection	Forefoot post-operative infection	Mixed chromatogram	*Peptinophilus harei*	*Peptinophilus harei*	Not tested
				*Anaerococcus murdochii*	*Streptococcus agalactiae*	
				*Streptococcus agalactiae*	*Anaerococcus murdochii*	
26	Radius bone biopsy	Firearm injury	Mixed chromatogram	*Streptococcus mitis/oralis/pneumoniae*	*Corynebacterium accolens/kroppenstedtii/* *tuberculostearicum*	Not tested
				*Staphylococcus epidermidids/capitis*	*Serratia quinovorans/liquefaciens*	
				*Comamonas* spp.	*Klebsiella* spp.	
				*Mycoplasma ovale*	*Aliidiomarina* spp.	
				*Aquabacterium* spp.	*Streptococcus* spp.	
				*Acinetobacter* spp.	*Veillonella* spp.	
				*Acidovorax* spp.	*Capnocytophaga* spp.	
					*Pseudomonas* spp.	
					*Microbacterium* spp.	
					*Staphylococcus* spp.	
27	Bone	Hand osteo-arthritis following a human bite	Mixed chromatogram (possible presence of *Fusobacterium* spp.)	*Fusobacterium nucleatum/canelifelinum*	*Fusobacterium nucleatum/canelifelinum*	Not tested
				*Tannerella forsythia*	*Tannerella forsythia*	
					*Bacteroides pyogenes*	
28	Brain abscess	Parieto-occipital brain abscess without portal of entry detected	Mixed chromatogram	*Campylobacter gracilis/showae/hominis*	*Schaalia meyeri*	Not tested
				*Neisseria elongata*	*Fusobacterium nucleatum*	
				*Fusobacterium nucleatum*	*Campylobacter rectus/gracilis/showae*	
				*Schaalia meyeri*	*Neisseria artica*	
				*Eikenella corrodens*	*Kingella denitrificans*	
				*Kingella denitrificans*	*Actinomyces israelii*	
				**Full-length 16S rRNA (sorted by decreasing abundance)**	**In-house partial 16S rRNA (sorted by decreasing abundance)**	
				**MinION regular flow-cell run**	**MinION regular flow-cell run**	**MinION Flongle run**
29	Mandible bone biopsy	NA	Mixed chromatogram (possible presence of *Porphyromonas* spp.)	*Fusobacterium nucleatum*	*Porphyromonas gingivalis*	Not tested
				*Porphyromonas gingivalis*	*Fusobacterium nucleatum*	
				*Filifactor alocis*	*Tannerella forsythia*	
				*Parvimonas micra*	*Filifactor alocis*	
				*Tannerella forsythia*	*Staphylococcus aureus/haemolyticus/simiae*	
30	Tibial bone biopsy	NA	Mixed chromatogram	*Streptococcus sanguinis/mitis/parasanguinis*	*Streptococcus sanguinis/mitis/oralis/* *pseudopneumoniae*	Not tested
				*Haemophilus parainfluenzae*	*Lactococcus lactis*	
				*Lactococcus lactis*	*Haemophilus parainfluenzae*	
					*Rothia mucilaginosa*	
					*Prevotella* spp.	
					*Neisseria macacae/perflava*	
					*Acitnomyces oris*	
					*Capnocytophaga* spp.	
					*Veillonella* spp.	
					*Porphyromonas gingivalis*	
31	Pleural aspirate	Pleuropneumonia	Mixed chromatogram	*Campylobacter rectus/showae*	*Fusobacterium nucleateum*	Not tested
				*Fusobacterium nucleateum*	*Treponema lecithinolyticum*	
				*Treponema lecithinolyticum*	*Porphyromonas gingivalis*	
				*Porphyromonas gingivalis*	*Tannerella forsythia*	
				*Tannerella forsythia*	*Campylobacter rectus*	

*NA, not-available*.

*No amplification: no amplification was detected after the full-length16S rRNA gene PCR*.

### Comparative Performance of Partial and Full-Length 16S rRNA Amplification

**Figure 3:**
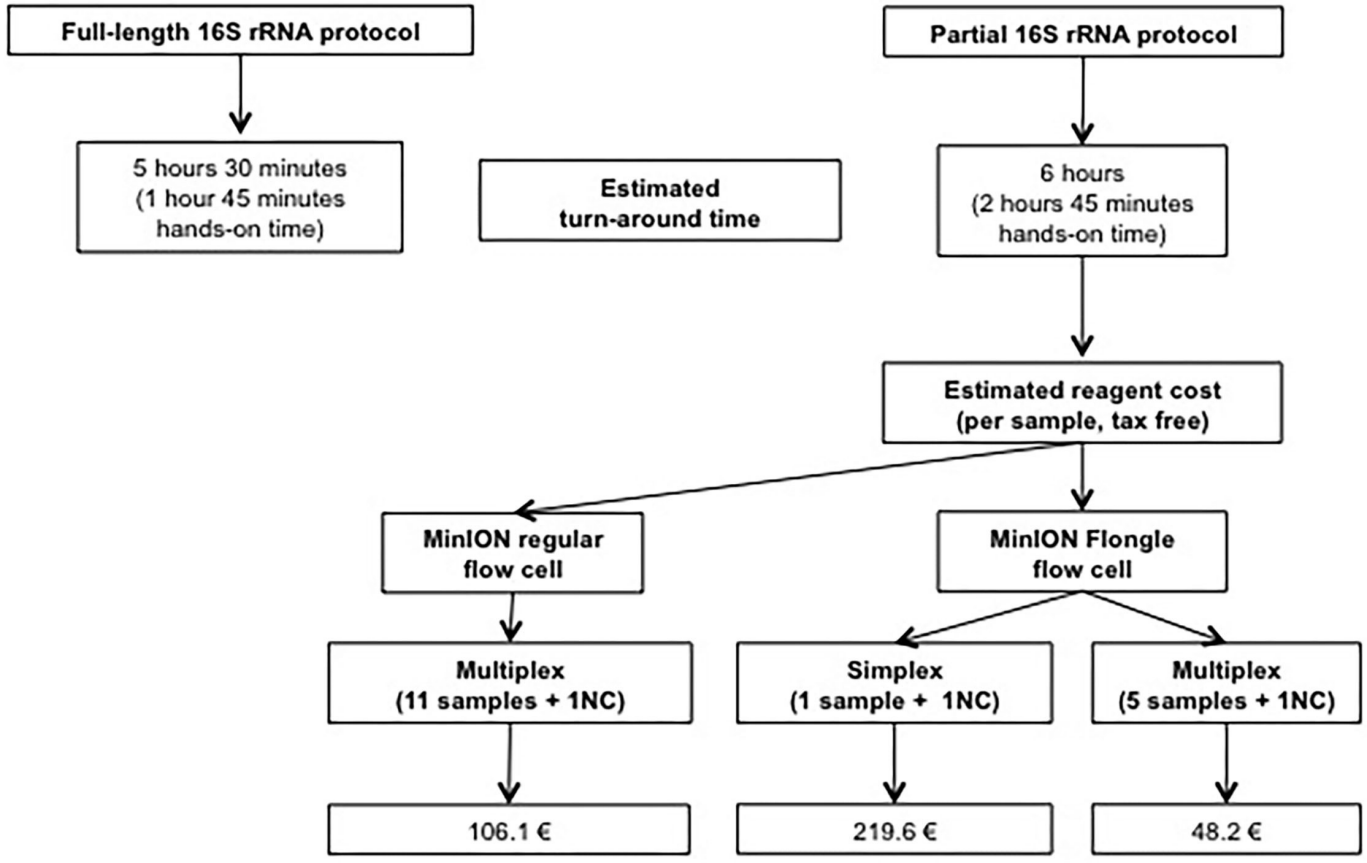
Comparison of Estimated Total Time and Reagent Cost of 16S rRNA Nanopore Technology.

Compared to the in-house partial 16S protocol, the full-length 16S protocol allowed both a shorter sample preparation time (5 h 30 min vs. 6 h) and a shorter hands-on time (1 h 45 min vs. 2 h 45 min; Figure 3).

Regarding sensitivity, the use of the full-length 16S rRNA barcoding kit allowed DNA amplification in only 24 of the 31 clinical samples; the remaining seven samples (samples 3–5, 11, 15, 16, and 19) did not yield a sufficient number of amplicons to be sequenced and further analyzed (Table 1). Of the positive samples, nine were found monomicrobial and with the same bacterial identification between the two approaches (Table 1). Noticeably, sample 12 was detected as monomicrobial with *Streptococcus agalactiae* using the full-length 16S protocol, while the partial 16S approach concluded the presence of both *Streptococcus agalactiae* and *Porphyromonas endodontalis*. The remaining 14 positive samples were categorized as polymicrobial with the same predominant bacteria using both the partial and full-length 16S protocol, even though not systematically ranked in the same order. Only samples 23 and 26 led to discordant bacterial identifications between the two approaches, with no overlap of the top-ranked bacteria (Table 1). Of note, the amplification signals were very weak, and the sequencing run yielded a majority of unclassified short fragments, probably primer dimers, leading to a difficult interpretation; these two observations strongly support the lack of clinical relevance.

### Cost

Per sample cost was 106.1€ for a 12-plex regular flow cell (Figure 3). In order to improve cost-effectiveness, a switch to the miniaturized flow cell (Flongle) and multiplexing was considered. Multiplexing up to five samples (samples 1–5) plus a negative control was successful, yielding the same results for each sample (Table 1). This allowed dropping the cost to 48.2€/sample when switching to a 6-plex Flongle flow cell (Figure 3).

## Discussion

We assessed herein the performance and issues of a targeted 16S rRNA metagenomic approach using Nanopore sequencing on culture-negative mono- and polybacterial clinical samples, and compared the in-house 16S partial amplification with the full-length 16S dedicated Nanopore kit. The cost was also evaluated in order to implement the technology as a routine diagnostic procedure.

Addressing the widely discussed issue of background noise (Street et al., 2017; Sanderson et al., 2018; Charalampous et al., 2019; Yang et al., 2019; Chan et al., 2020), we found herein that setting a cut-off of 1% classified reads improved the accuracy of results, in line with many published studies (Sanderson et al., 2018; Charalampous et al., 2019; Yang et al., 2019; Chan et al., 2020). Nonetheless, using this cut-off in sample 31, collected from pleuropneumonia, led to categorising some bacteria as background noise while these are well-known oral flora bacteria (Nakazawa et al., 2002; Heintz et al., 2019). Conversely, the analysis of sample 22 (aortic valve in a context of infective endocarditis) concluded with the notable presence of six bacteria including environmental and oral flora bacteria, questioning their relevance as etiological agents. This highlights the challenge of identifying the true infective pathogen and the need for expertise by a skilled professional, on top of standard cut-offs.

Regarding identification accuracy, we confirmed the agreement between Nanopore and Sanger-based sequencing, which is in line with previous studies (Moon et al., 2017; Ashikawa et al., 2018; Cheng et al., 2018). The well-known major drawback of 16S rRNA gene sequencing relies on sequence similarities inducing poor discriminatory power at the species-level, and even at the genus-level for some bacterial clades. Jonhson et al. have shown that taxonomic resolution depends on the length of the analyzed 16S rRNA gene fragment and the chosen sub-region (Johnson et al., 2019). We here found a satisfactory balance between sensitivity of detection and accuracy of bacterial identification. Nonetheless, it should be reminded that, although often used for species-level identification (Yang et al., 2019; Chan et al., 2020), the Epi2Me pipeline was designed for genus-level identification only (manufacturer's recommendations) and should therefore be used with caution otherwise.

Cost is a major issue in metagenomics; we addressed this herein and found that Nanopore sequencing was a competitive option; the per-sample cost was 69.6€ for a 12-plex MinION regular flow-cell run and 48.2€ for a 6-plex Flongle run. In addition, we compared both partial and full-length 16S approaches and found that the latter offered a more user-friendly library preparation protocol with reduced hands-on time. However, the substantially greater sensitivity of the in-house partial 16S rRNA amplification, probably linked to the amplification of a shorter DNA fragment, prompted us to adopt the in-house protocol. The development of a kit using the partial 16S rRNA amplification could lead to a user-friendly and sensitive system.

Taken together, and from a clinical application point of view, herein, Nanopore sequencing allowed the identification of the bacteria present in both monomicrobial and polymicrobial samples, resolving the dead-end microbiological diagnoses. Bacterial identifications were consistent with well-known aetiologies (Plainvert et al., 2012; Selton-Suty et al., 2012; Vieira et al., 2015; Dyrhovden et al., 2019) but also scarcely reported aetiologies (Martiny et al., 2017), opening the way for the recognition of new aetiologies. In conclusion, Nanopore 16S rRNA targeted metagenomics appeared as a user-friendly, de-skilled, cost- and time-effective tool for clinical laboratories to ensure the routine diagnosis of culture-negative samples. This study paves the way for further prospective studies that will explore the clinical impact of this approach used in routine practise.

## Data Availability Statement

The datasets presented in this study can be found in online repositories. The names of the repository/repositories and accession number(s) can be found below: https://www.ebi.ac.uk/ena, PRJEB41337.

## Ethics Statement

This project was conducted according to the French law in force at the time of the study and ethical principles outlined in the Declaration of Helsinki. It obtained approval by the institutional Ethics Committee (number 20-265). To be included in this research the patients gave a “non-opposition” form in line with French regulation.

## Author Contributions

CB was given the grant, designed and performed the study, and wrote the manuscript. CG helped design the study, trained CB in the Nanopore technology, provided technical expertise, and helped write the manuscript. YB performed part of the analyses and reviewed the manuscript. TG, HS, and OD helped designing the study and reviewing the manuscript. FL helped to obtain the grant, designing the study, and reviewing the manuscript. FV wrote the manuscript and helped to design the study. FV and FL gave medical input and expertise on the strategy of Nanopore technology development on a routine basis. All authors contributed to the article and approved the submitted version.

## Funding

This work was supported by the Hospices Civils de Lyon with the Grant Innovation Award 2019 although funders had no role in study design, data collection, and interpretation.

## Conflict of Interest

The authors declare that the research was conducted in the absence of any commercial or financial relationships that could be construed as a potential conflict of interest.

## Publisher's Note

All claims expressed in this article are solely those of the authors and do not necessarily represent those of their affiliated organizations, or those of the publisher, the editors and the reviewers. Any product that may be evaluated in this article, or claim that may be made by its manufacturer, is not guaranteed or endorsed by the publisher.
