# Supplementary material for: Improving the Diagnosis of Bacterial Infections: Evaluation of 16S rRNA Nanopore Metagenomics in Culture-Negative Samples
Source: Front Microbiol. 2022 Jul 14;13:943441. doi: 10.3389/fmicb.2022.943441 (PMC9329087; doi:10.3389/fmicb.2022.943441)
Supplement: Supplementary file 1 [file Table_1.docx]

Supplementary Table 1. Characteristics and bacterial identifications based on Sanger sequencing and in-house partial 16S rRNA Nanopore sequencing. Identifications and corresponding percent abundance above the 1% cut-off are indicated in bold type.

| Sample ID | Sample type | Clinical context | Sanger-based sequencing | Nanopore-based sequencing ID  (top ten bacteria) | Reads | % |
| --- | --- | --- | --- | --- | --- | --- |
|  |  |  |  | In-house partial 16S rRNA approach |  |  |
| 1 | Bone | NA | *Haemophilus parainfluenzae* | ***Haemophilus parainfluenzae***  *Tatumella terrea*  *Pseudoalteromonas spp*  *Corynebacterium accolens/minutissimum*  *Actinobacillus arthitidis*  *Alteromonas spp*  *Vibrio spp*  *Streptococcus salivarius*  *Serratia spp*  *Zymobacter spp*  Other (41)  Total | 16711  26  21  17  8  8  8  7  6  6  171  16989 | ***98.36***  *0.16*  *0.12*  *0.10*  *0.05*  *0.05*  *0.05*  *0.04*  *0.04*  *0.04*  *1.01*  *100.00* |
| 2 | Bone | Chronic bone abscess | *Clostridium spp* | ***Clostridium massilodielmoense***  *Tannerella forsythia*  *Fusobacterium nucleatum*  *Pseudonocardia acaciae*  *Bacteroides pyogenes*  *Porphyromonas gulae*  *Corynebacterium kroppenstedtii*  *Streptococcus fryi*  *Klebsiella pneumoniae*  *Bacillus mobilis*  Other (6)  Total | *7221*  *72*  *66*  *8*  *4*  *2*  *2*  *1*  *1*  *1*  *6*  *7438* | ***97.08***  *0.97*  *0.89*  *0.11*  *0.05*  *0.03*  *0.03*  *0.01*  *0.01*  *0.01*  *0.08*  *100.00* |
| 3 | Bone | Chronic osteomyelitis | *Enterobacteriaceae* | ***Salmonella enterica***  ***Streptococcus mitis/oralis/pseudopneumoniae***  ***Moraxella osloensis***  ***Haemophilus parainfluenzae***  ***Chryseobacterium spp***  ***Staphylococcus pasteuri***  *Gemella haemolysans*  *Neisseria spp*  *Granulicatella elegans/adiacens*  *Rothia mucilaginosa*  Other (57)  Total | *3560*  *1232*  *610*  *203*  *102*  *77*  *58*  *41*  *23*  *20*  *218*  *5906* | ***60.28***  ***20.86***  ***10.33***  ***3.44***  ***1.73***  ***1.30***  *0.98*  *0.69*  *0.39*  *0.34*  *3.69*  *100.00* |
| 4 | Bone abscess | Sarcoma | *Bacillus spp* | ***Bacillus mobilis***  *Cutibacterium acnes*  *Bacteroides uniformis*  *Sporolactobacillus spp*  *Oscillibacter spp*  *Lactobacillus spp*  *Bifidobacterium spp*  *Paenibacillus spp*  *Halobacillus spp*  *Alistipes spp*  Other (38)  Total | *9593*  *47*  *19*  *15*  *9*  *8*  *5*  *5*  *4*  *4*  *65*  *9774* | ***98.15***  *0.48*  *0.19*  *0.15*  *0.09*  *0.08*  *0.05*  *0.05*  *0.04*  *0.04*  *0.67*  *100.00* |
| 5 | Bone | Hip prosthesis infection | *Klebsiella pneumoniae* | ***Klebsiella pneumoniae***  *Enterobacter cloacae/asburiae*  *Staphylococcus aureus/simiae*  *Serratia marcescens*  *Pectobacterium polaris*  *Tatumella terrea*  *Cutibacterium acnes*  *Shimwellia spp*  *Xenorhabdus spp*  *Citrobacter spp*  Other (42)  Total | *21112*  *169*  *136*  *108*  *57*  *32*  *21*  *17*  *12*  *11*  *78*  *21753* | ***97.05***  *0.78*  *0.63*  *0.50*  *0.26*  *0.15*  *0.10*  *0.08*  *0.06*  *0.05*  *0.36*  *100.00* |
| 6 | Aortic valve | Infective endocarditis | *Streptococcus gallolyticus* | ***Streptococcus gallolyticus***  *Corynebacterium kroppenstedtii*  *Lactococcus spp*  *Lactobacillus spp*  *Enterococcus faecium*  *Haemophilus parainfluenzae*  *Clostridium massiliodielmoense*  *Cutibacterium acnes*  *Carnobacterium spp*  *Leuconostoc spp*  Other (20)  Total | *17563*  *114*  *14*  *14*  *6*  *4*  *3*  *2*  *2*  *2*  *23*  *17747* | ***98.96***  *0.64*  *0.08*  *0.08*  *0.03*  *0.02*  *0.02*  *0.01*  *0.01*  *0.01*  *0.13*  *100.00* |
| 7 | Bone | Tibial external fixators infection | *Staphylococcus spp* | ***Staphylococcus aureus /lugdunensis/ simiae***  *Macrococcus spp*  *Bacillus spp*  *Jeotgalicoccus spp*  *Corynebacterium accolens*  *Enterococcus spp*  *Pseudomonas stutzeri*  *Vagococcus penaei*  *Listeria riparia*  *Salinococcus spp*  Other (56)  Total | *53146*  *411*  *98*  *85*  *48*  *20*  *20*  *17*  *14*  *13*  *135*  *54007* | ***98.41***  *0.76*  *0.18*  *0.16*  *0.09*  *0.04*  *0.04*  *0.03*  *0.03*  *0.02*  *0.25*  *100.00* |
| 8 | Aqueous humor | Post cataract-surgery endophtalmitis | *Streptococcus spp* | ***Streptococcus oralis / himalayensis / parasanguinis / pneumoniae***  *Enterococcus saccharolyticus*  *Lactobacillus spp*  *Carnobacterium spp*  *Lactococcus spp*  *Pseudomonas stutzeri*  *Marinilactibacillus spp*  *Corynebacterium kroppenstedtii*  *Alkalibacterium spp*  *Paenibacillus spp*  Other (56)  Total | *49156*  *142*  *39*  *32*  *29*  *29*  *28*  *15*  *12*  *12*  *122*  *49616* | ***99.07***  *0.29*  *0.08*  *0.06*  *0.06*  *0.06*  *0.06*  *0.03*  *0.02*  *0.02*  *0.21*  *100.00* |
| 9 | Liver abscess | Liver abscess returning from Algeria trip | *Klebsiella spp* | ***Klebsiella spp***  *Pectobacterium polaris*  *Tatumella spp*  *Shimwellia spp*  *Xenorhabdus spp*  *Spiribacter spp*  *Gibbsiella spp*  *Streptococcus spp*  *Marinomonas spp*  *Mobilococcus spp*  Other (52)  Total | *41624*  *404*  *68*  *54*  *14*  *14*  *13*  *9*  *8*  *7*  *146*  *42361* | ***98.26***  *0.95*  *0.16*  *0.13*  *0.03*  *0.03*  *0.03*  *0.02*  *0.02*  *0.02*  *0.34*  *100.00* |
| 10 | Pleural aspirate | Pleuropneumonia | *Streptococcus pyogenes* | ***Streptococcus pyogenes***  *Enterococcus faecium*  *Lactobacillus spp*  *Lactococcus spp*  *Brevibacillus spp*  *Pseudomonas stutzeri*  *Carnobacterium viridans*  *Pectobacterium polaris*  *Paenibacillus spp*  *Kurthia spp*  Other (96)  Total | *262050*  *146*  *135*  *128*  *83*  *66*  *51*  *39*  *26*  *24*  *225*  *262973* | ***99.65***  *0.06*  *0.05*  *0.05*  *0.03*  *0.03*  *0.02*  *0.01*  *0.01*  *0.01*  *0.09*  *100.00* |
| 11 | Tissue from bone and joint infection | NA | Polybacterial pattern chromatogram | ***Streptococcus dysgalactiae***  *Vibrio fluvialis*  *Staphylococcus epidermidis/hominis*  *Veillonella ratti*  *Porphyromonas endodontalis*  *Cutibacterium acnes*  *Micrococcus spp*  *Enterococcus spp*  *Corynebacterium spp*  *Lactobacillus spp*  Other (43)  Total | *17087*  *107*  *69*  *69*  *69*  *45*  *39*  *34*  *33*  *28*  *126*  *17706* | ***96.50***  *0.59*  *0.39*  *0.39*  *0.39*  *0.25*  *0.22*  *0.19*  *0.19*  *0.16*  *0.71*  *100.00* |
| 12 | Soft tissue biopsy | NA | Polybacterial pattern chromatogram | ***Streptococcus agalactiae***  *Porphyromonas endodontalis*  *Fusobacterium nucleatum*  *Lactobacillus spp*  *Enterococcus spp*  *Carnobacterium spp*  *Anoxybacillus spp*  *Tannerella forsythia*  *Aerococcus urinae*  *Alkalibacterium spp*  Other (40)  Total | *27263*  ***333***  *122*  *37*  *32*  *23*  *12*  *10*  *10*  *9*  *94*  *27945* | ***97.56***  ***1.19***  *0.44*  *0.13*  *0.11*  *0.08*  *0.04*  *0.04*  *0.04*  *0.03*  *0.34*  *100.00* |
| 13 | Disc biopsy | Spondylodiscitis | Polybacterial pattern chromatogram (possible presence of *Staphylocococcus* spp) | ***Staphylococcus epidermidis/saccharolyticus***  *Jeotgalicoccus halotolerans*  *Bacillus spp*  *Macrococcus spp*  *Burkholderia spp*  *Paenibacillus spp*  *Streptococcus oralis*  *Lactobacillus spp*  *Listeria marthii*  *Vagogoccus penaei*  Other (14)  Total | *9977*  *17*  *15*  *8*  *5*  *3*  *2*  *2*  *2*  *2*  *14*  *10051* | ***99.26***  *0.17*  *0.15*  *0.08*  *0.05*  *0.03*  *0.02*  *0.02*  *0.02*  *0.02*  *0.14*  *100.00* |
| 14 | Elbow joint fluid | NA | Polybacterial pattern chromatogram | ***Staphylococcus simiae/aureus/haemolyticus***  *Macrococcus bohemicus*  *Bacillus spp*  *Jeotgalicoccus spp*  *Streptococcus parasangunis*  *Vagococcus martis*  *Cutibacterium acnes*  *Paracoccus marinus*  *Halobacillus spp*  *Listeria riparia*  Other (4)  Total | *4024*  *18*  *7*  *6*  *4*  *2*  *2*  *2*  *1*  *1*  *4*  *4069* | ***98.89***  *0.44*  *0.17*  *0.15*  *0.09*  *0.05*  *0.05*  *0.05*  *0.02*  *0.02*  *0.10*  *100.00* |
| 15 | Reaming product | Hip prosthesis infection | Polybacterial pattern chromatogram | ***Cutibacterium acnes***  ***Burholderia diffusa***  *Paenibacillus thermoaerophilus*  *Nocardioides spp*  *Acidipropionibacterium spp*  *Corynebacterium spp*  *Streptosporangium spp*  *Rhodococcus rhodnii*  *Gordonia spp*  *Rhizobium spp*  Other (85)  Total | *55079*  *1018*  *304*  *36*  *17*  *16*  *13*  *12*  *8*  *8*  *227*  *56738* | ***97.08***  ***1.79***  *0.53*  *0.06*  *0.03*  *0.03*  *0.02*  *0.02*  *0.01*  *0.01*  *0.41*  *100.00* |
| 16 | Aqueous humor | Endophtalmitis | Polybacterial pattern chromatogram | ***Raoultella spp***  *Pectobacterium polaris*  *Tatumella punctate*  *Marinomonas spp*  *Pseudoalteromonas spp*  *Spiribacter curvatus*  *Xenorhabdus spp*  *Gibbsiella spp*  *Rosenbergiella spp*  *Yokenella spp*  Other (84)  Total | *103080*  *805*  *444*  *52*  *51*  *27*  *22*  *12*  *12*  *12*  *264*  *104781* | ***98.40***  *0.77*  *0.42*  *0.05*  *0.05*  *0.03*  *0.02*  *0.01*  *0.01*  *0.01*  *0.25*  *100.00* |
| 17 | Joint fluid | NA | Polybacterial pattern chromatogram | ***Chryseobacterium spp***  ***Acinetobacter parvus/septicus***  ***Paracoccus lutimaris***  ***Moraxella osloensis***  ***Microbacterium lacticum***  ***Haematobacter massiliensis***  ***Pseudoxanthomonas spp***  *Ochrobactrum spp*  *Photobacterium kishitanii*  *Sphingobium scionense*  Other (41)  Total | *4636*  *2786*  *2319*  *1388*  *502*  *408*  *368*  *78*  *63*  *62*  *290*  *12900* | ***35.94***  ***21.60***  ***18.00***  ***10.76***  ***3.89***  ***3.16***  ***2.85***  *0.60*  *0.49*  *0.48*  *2.25*  *100.00* |
| 18 | Bone | Sacroileitis | Polybacterial pattern chromatogram | ***Porphyromonas endodontalis/gingivalis,***  ***Streptococcus pseudopneumoniae/parasanguinis***  ***Staphylococcus hominis/epidermidis/saccharolyticus,***  ***Veillonella ratti/tobetsuensis,***  ***Corynebacterium lipophiloflavum/pilbarense,***  ***Micrococcus spp,***  ***Cutibacterium spp,***  ***Dermacoccus spp***  ***Rothia mucilaginosa***  ***Finegoldia magna***  Other (75)  Total | *2647*  *2438*  *2292*  *1769*  *1283*  *1125*  *376*  *316*  *254*  *193*  *231*  *12924* | ***20.48***  ***18.86***  ***17.73***  ***13.69***  ***9.93***  ***8.70***  ***2.90***  ***2.45***  ***1.97***  ***1.49***  *1.79*  *100.00* |
| 19 | Soft tissue biopsy | Deep-seated soft tissue infection | Polybacterial pattern chromatogram | ***Corynebacterium kroppenstedtii/tuberculostearicum***  ***Morganelle morganii***  ***Anaerococcus octavus/nagyae***  ***Cutibacterium acnes***  ***Staphylococcus epidermidis/pasteurii/capitis***  ***Prevotella veroralis/maculosa***  ***Streptococcus parasanguinis/gordonii***  ***Actinomyces oris***  ***Finegoldia magna***  ***Stenotrophomonas koreensis***  Other (51)  Total | *581*  *352*  *342*  *312*  *274*  *251*  *231*  *183*  *149*  *147*  *777*  *3599* | ***16.14***  ***9.78***  ***9.50***  ***8.67***  ***7.53***  ***6.97***  ***6.42***  ***5.08***  ***4.14***  ***4.08***  *21.59*  *100.00* |
| 20 | Thorax tissular biopsy | Firearm injury | Polybacterial pattern chromatogram | ***Bacteroides uniformis/vulgatus***  ***Bifidobacterium spp***  ***Oscillibacter valericigenes***  ***Collinsella aerofaciens***  ***Paenibacillus spp***  ***Ruminococcus spp***  ***Hungateiclostridium spp***  ***Dialister invisus***  ***Parvimonas micra***  ***Alistipes putredinis***  Other (64)  Total | *702*  *310*  *258*  *234*  *151*  *123*  *85*  *80*  *76*  *62*  *470*  *2551* | ***27.52***  ***12.15***  ***10.11***  ***9.17***  ***5.91***  ***4.82***  ***3.33***  ***3.14***  ***2.98***  ***2.43***  *18.42*  *100.00* |
| 21 | Pleural aspirate | Pleuropneumonia | Polybacterial pattern chromatogram | ***Porphyromonas gingivalis***  ***Treponema maltophilum/denticola/medium***  ***Fusobacterium nucleatum***  ***Filifactor alocis***  ***Tannerella forsythia***  ***Schaalia cardiffensis***  ***Mogibacterium timidum***  ***Prevotella seregens***  *Slackia exigua*  *Peptococcus niger*  Other (95)  Total | *11173*  *7045*  *5574*  *3022*  *1887*  *1094*  *813*  *325*  *176*  *150*  *429*  *31688* | ***35.26***  ***22.23***  ***17.59***  ***9.54***  ***5.95***  ***3.45***  ***2.57***  ***1.03***  *0.56*  *0.47*  *1.35*  *100.00* |
| 22 | Aortic valve | Infective endocarditis | Polybacterial pattern chromatogram | ***Cutibacterium acnes***  ***Enterobacter asburiae/cloacae***  ***Stenotrophomonas pavanii/hibiscicola***  ***Chryseobacterium spp***  ***Moraxella spp***  ***Streptococcus mitis/oralis***  *Paracoccus lutimaris*  *Microbacterium lacticum*  *Atopobium parvulum*  *Pectobacterium spp*  Other (68)  Total number | *6913*  *3193*  *167*  *154*  *142*  *124*  *87*  *34*  *17*  *11*  *198*  *11040* | ***62.62***  ***28.92***  ***1.51***  ***1.39***  ***1.29***  ***1.12***  *0.79*  *0.31*  *0.15*  *0.10*  *1.79*  *100.00* |
| 23 | Vascular tissue | Vascular prosthesis infection in a context of lumbosacral eschar | Mixed chromatogram | ***Cloacibacterium normanense***  ***Moraxella osloensis***  ***Prevotella spp***  ***Pleomorphomonas oryzae***  ***Tolumonas osonensis***  ***Aeromonas schubertii***  ***Bacteroides graminisolvens***  ***Geobacillus stearothermophilus***  ***Acinetobacter johnsonii***  ***Brachybacterium paraconglomeratum***  Other (51)  Total number | *4282*  *1338*  *505*  *431*  *373*  *364*  *275*  *222*  *218*  *216*  *863*  *9087* | ***47.12***  ***14.72***  ***5.56***  ***4.74***  ***4.10***  ***4.01***  ***3.03***  ***2.44***  ***2.40***  ***2.38***  *9.50*  *100.00* |
| 24 | Spleen abscess | NA | Mixed chromatogram | ***Bacteroides fragilis***  ***Porphyromonas endodontalis***  ***Streptococcus spp***  ***Treponema lecithinolyticum***  ***Haemophilus parainfluenzae***  ***Bacillus mobilis***  ***Klebsiella pneumoniae***  ***Staphylococcus epidermidis/haeomolyticus***  *Methyloversatilis spp*  *Clostridium massiliodielmoense*  Other (4)  Total number | *1533*  *1352*  *102*  *100*  *82*  *51*  *48*  *44*  *21*  *20*  *41*  *3394* | ***45.17***  ***39.84***  ***3.01***  ***2.95***  ***2.42***  ***1.50***  ***1.41***  ***1.30***  *0.62*  *0.59*  *1.21*  *100.00* |
| 25 | Tissue from bone and joint infection | Forefoot post-operative infection | Mixed chromatogram | ***Peptinophilus harei***  ***Streptococcus agalactiae***  ***Anaerococcus murdochii***  *Corynebacterium jekeium*  *Staphylococcus nepalensis/epidermidis*  *Lactobacillus spp*  *Lactococcus spp*  *Enterococcus faecium*  *Pseudomonas stutzeri*  *Clostridium oceanicum*  Other (9)  Total | *2243*  *1805*  *1506*  *7*  *6*  *4*  *2*  *2*  *1*  *1*  *12*  *5589* | ***40.13***  ***32.30***  ***26.95***  *0.13*  *0.11*  *0.07*  *0.04*  *0.04*  *0.02*  *0.02*  *0.21*  *100.00* |
| 26 | Radius bone biopsy | Firearm injury | Polybacterial pattern chromatogram | ***Corynebacterium accolens/kroppenstedtii/tuberculostearicum***  ***Serratia quinovorans/liquefaciens***  ***Klebsiella spp***  ***Aliidiomarina spp***  ***Streptococcus spp***  ***Veillonella spp***  ***Capnocytophaga spp***  ***Pseudomonas spp***  ***Microbacterium spp***  ***Staphylococcus spp***  Other (42)  Total | *6430*  *1354*  *1102*  *1083*  *897*  *813*  *697*  *522*  *304*  *284*  *512*  *13998* | ***45.94***  ***9.67***  ***7.87***  ***7.74***  ***6.41***  ***5.81***  ***4.98***  ***3.73***  ***2.17***  ***2.03***  *3.66*  *100.00* |
| 27 | Bone | Hand osteo-arthritis following a human bite | Polybacterial pattern chromatogram (possible presence of *Fusobacterium spp*) | ***Fusobacterium nucleatum/canelifelinum***  ***Tannerella forsythia***  ***Bacteroides pyogenes***  *Corynebacterium kroppenstedtii*  *Lactococcus lactis*  *Porphyromonas gulae*  *Methylobacterium spp*  *Methylorubrum spp*  *Parabacteroides merdae*  *Sreptococcus agalactiae*  Other (8)  Total | *4585*  *3533*  *115*  *59*  *46*  *30*  *8*  *7*  *3*  *2*  *8*  *8396* | ***54.61***  ***42.08***  ***1.37***  *0.70*  *0.55*  *0.36*  *0.10*  *0.08*  *0.04*  *0.02*  *0.10*  *100.00* |
| 28 | Brain abscess | Parieto-occipital brain abscess without portal of entry detected | Polybacterial pattern chromatogram | ***Schaalia meyeri***  ***Fusobacterium nucleatum***  ***Campylobacter rectus/gracilis/showae***  ***Neisseria artica***  ***Kingella denitrificans***  ***Actinomyces israelii***  *Conchiformibius kuhniae*  *Alysiella filiformis*  *Vogesella amnigena*  *Arcanobacterium spp*  Other (168)  Total | *34498*  *11329*  *9083*  *4452*  *1226*  *1135*  *160*  *153*  *97*  *29*  *636*  *62798* | ***54.93***  ***18.04***  ***14.46***  ***7.09***  ***1.95***  ***1.81***  *0.25*  *0.24*  *0.15*  *0.05*  *1.01*  *100.00* |
| 29 | Mandible bone biopsy | NA | Mixed chromatogram (possible presence of *Porphyromonas* *spp*) | ***Porphyromonas gingivalis***  ***Fusobacterium nucleatum***  ***Tannerella forsythia***  ***Filifactor alocis***  ***Staphylococcus simiae/aureus/haemolyticus***  *Parvimonas micra*  *Desulfobulbus mediterraneus*  *Treponema denticola*  *Kineothrix alysoides*  *Streptococcus massiliensis*  Other (16)  Total | *10469*  *4016*  *417*  *254*  *155*  *123*  *36*  *33*  *15*  *10*  *42*  *15570* | ***67.24***  ***25.79***  ***2.68***  ***1.63***  ***1.00***  *0.79*  *0.23*  *0.21*  *0.10*  *0.06*  *0.27*  *100.00* |
| 30 | Tibial bone biopsy | NA | Mixed chromatogram | ***Streptococcus sanguinis/mitis/oralis/pseudopneumoniae***  ***Lactococcus lactis***  ***Haemophilus parainfluenzae***  ***Rothia mucilaginosa***  ***Prevotella spp***  ***Neisseria macacae/perflava***  ***Acitnomyces oris***  ***Capnocytophaga spp***  ***Veillonella spp***  ***Porphyromonas gingivalis***  Other (228)  Total | *38332*  *24997*  *19638*  *5736*  *2829*  *2531*  *2086*  *2031*  *1605*  *1366*  *8326*  *111508* | ***34.38***  ***22.42***  ***17.61***  ***5.14***  ***2.54***  ***2.27***  ***1.87***  ***1.82***  ***1.44***  ***1.23***  *7.47*  *100.00* |
| 31 | Pleural aspirate | Pleuropneumonia | Mixed chromatogram | ***Fusobacterium nucleateum***  ***Treponema lecithinolyticum***  ***Porphyromonas gingivalis***  ***Tannerella forsythia***  ***Campylobacter rectus***  *Parvimonas micra*  *Mogibacterium diversum*  *Clostridium spp*  *Paenibacillus spp*  *Bacteroides spp*  Other (122)  Total | *68798*  *48132*  *20351*  *7168*  *1503*  *396*  *52*  *22*  *18*  *12*  *236*  *146688* | ***46.90***  ***32.81***  ***13.87***  ***4.89***  ***1.02***  *0.27*  *0.04*  *0.01*  *0.01*  *<0.01*  *0.16*  *100.00* |

NA: Not available
